# Pigmented Curvularia Keratitis Adjacent to a Bifid Pterygium: A Case Report

**DOI:** 10.7759/cureus.111012

**Published:** 2026-06-17

**Authors:** Pravalika Rebbala, Sathwik Reddy Nomula, Siddharam S Janti, Rahul Garg, Srividya Kalluri, Mounika Somasani, Sudha Arumugam, Vishweshwarraiah R Dongre

**Affiliations:** 1 Department of Ophthalmology, All India Institute of Medical Sciences, Bibinagar, Bibinagar, IND; 2 Department of Microbiology, All India Institute of Medical Sciences, Bibinagar, Bibinagar, IND

**Keywords:** bifid pterygium, curvularia, keratitis, pigmented corneal ulcer, topical steroid use

## Abstract

Fungal keratitis remains a major cause of corneal morbidity in the tropics, with dematiaceous fungi such as *Curvularia* representing an important subset. We report a case of *Curvularia* keratitis in a 55-year-old woman who presented with post-traumatic keratitis and prior use of topical corticosteroids. Examination revealed a bifid pterygium and a grayish-white stromal infiltrate with feathery margins located just anterior to the pterygium head. KOH mount and culture confirmed *Curvularia* species. The patient responded well to topical natamycin. This case underscores the importance of initiating early microbiological evaluation in suspected fungal keratitis, especially in steroid-exposed eyes.

## Introduction

Fungal keratitis is a significant cause of corneal blindness worldwide, especially in tropical and subtropical regions, where agricultural and outdoor activities increase the risk of ocular trauma [1,2]. Despite the reported incidence of *Curvularia *keratitis, its occurrence in association with a bifid pterygium is exceedingly rare. These organisms possess melanin in their cell walls, which enhances resistance to oxidative stress and contributes to virulence [3]. Clinically, dematiaceous fungal keratitis can present with brown or pigmented stromal infiltrates due to melanin deposition, although this feature may be easily overlooked [4]. The use of topical corticosteroids prior to definitive diagnosis is a recognized risk factor for worsening fungal keratitis, as it suppresses host immune responses and facilitates fungal proliferation [5]. We report a rare association of microbiologically proven *Curvularia *keratitis occurring adjacent to a bifid pterygium. The inflamed pterygial tissue may mask subtle stromal infiltrates, underscoring the need for careful slit-lamp examination to avoid overlooking fungal keratitis and inappropriate steroid use.

## Case presentation

A 55-year-old farmer presented with chief complaints of redness, pain, watering, and foreign body sensation in the left eye for one week following minor ocular trauma sustained with vegetative matter. Prior to presentation, she was prescribed topical 0.5% loteprednol four times daily for one week by a registered medical practitioner. She had no history of diabetes mellitus, immunosuppression, or previous ocular surgery.

**Figure 1:**
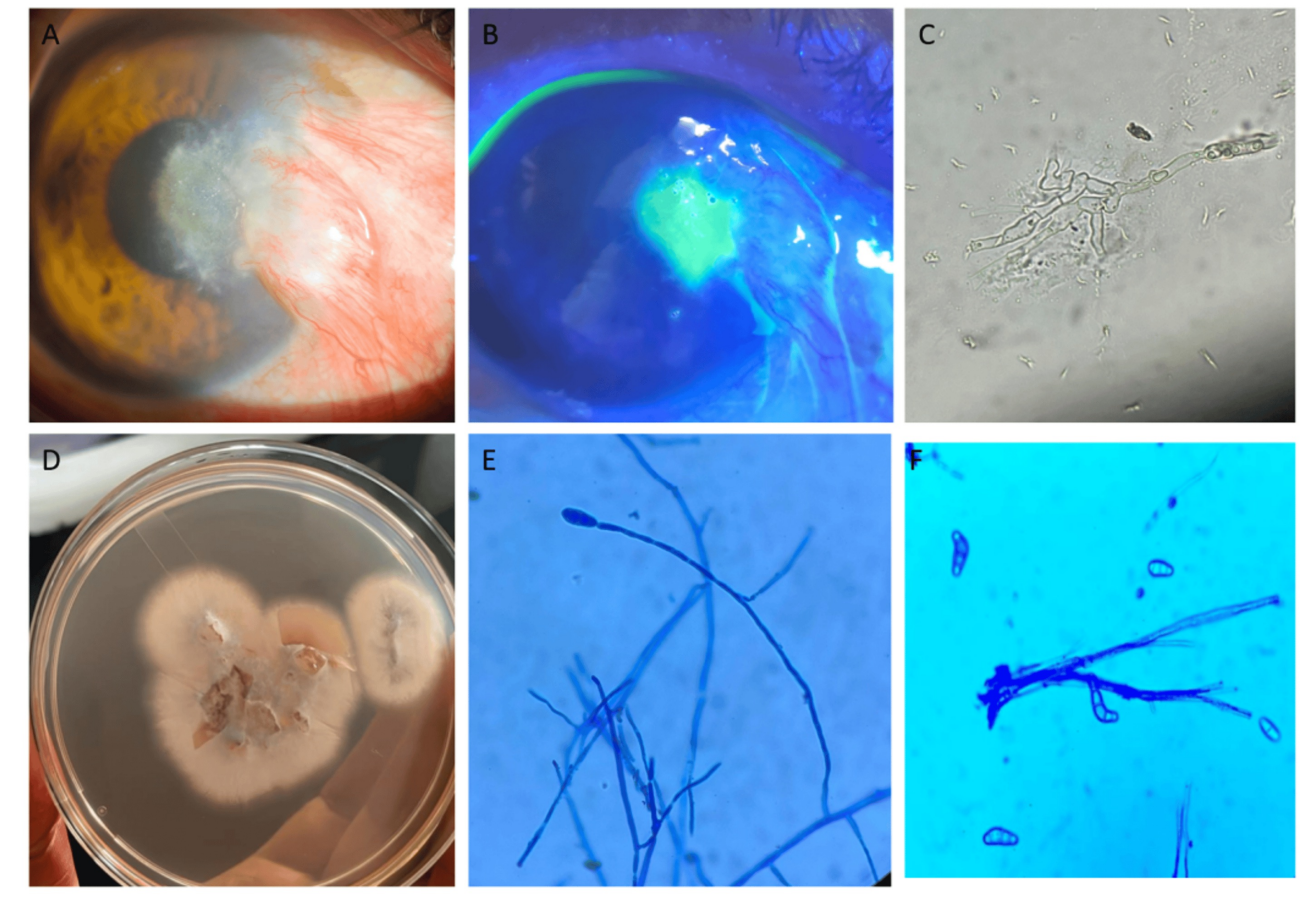
Clinical, microbiological, and mycological features of pigmented Curvularia keratitis adjacent to a bifid pterygium (A) Slit-lamp photograph showing conjunctival congestion with a bifid pterygium and a grayish-white corneal infiltrate with feathery margins and a dry surface noted just anterior to the pterygium head. (B) Fluorescein staining highlighting an epithelial defect overlying the corneal infiltrate. (C) Potassium hydroxide (KOH) wet mount of corneal scraping showing branching, septate fungal filaments. (D) Fungal culture on Sabouraud dextrose agar showing whitish-gray, velvety colonies. (E, F) Lactophenol cotton blue mount demonstrating *Curvularia *species with conidiophores bearing multicellular, slightly curved conidia.

On examination, best-corrected visual acuity was 6/6 in the right eye and 6/24 in the left eye. Slit-lamp examination of the left eye revealed circumcorneal congestion and a bifid pterygium extending from the temporal limbus onto the cornea. Just anterior to the pterygium head, a 1.5 × 2 mm paracentral grayish-white stromal infiltrate with feathery margins and a dry surface appearance with a central epithelial defect was noted (Figure 1A). A central epithelial defect measuring approximately 3 × 3 mm was noted on fluorescein staining (Figure 1B). Subtle brownish pigmentation was noted at the advancing edge of the infiltrate near the pupillary axis. A mild anterior chamber reaction (1+ cells) was present without hypopyon or stromal thinning. The right eye was unremarkable.

Corneal scrapings were obtained. Direct microscopy with a 10% KOH mount revealed branching, septate fungal filaments (Figure 1C). Culture on Sabouraud dextrose agar yielded whitish-gray, velvety colonies within three days. Lactophenol cotton blue staining demonstrated multicellular, slightly curved conidia consistent with *Curvularia *species (Figure 1D-1F).

Topical corticosteroid therapy was immediately discontinued. The patient was initiated on hourly topical 5% natamycin along with cycloplegic drops twice daily. Treatment was continued intensively during the initial days and subsequently tapered according to clinical response.

At one-week follow-up, the patient reported significant symptomatic improvement. Visual acuity improved to 6/12 in the affected eye. The epithelial defect and infiltrate both decreased in size to approximately 1 × 1 mm, with reduced stromal edema. The anterior chamber reaction had resolved. Antifungal therapy was continued at a reduced frequency. By two weeks, there was complete resolution of the active infiltrate with formation of a faint stromal scar. Final best-corrected visual acuity improved to 6/9, and no recurrence was noted on subsequent follow-up.

## Discussion

Dematiaceous fungi such as *Curvularia *are well-recognized causes of fungal keratitis in tropical climates, often associated with trauma involving vegetative matter [1,2]. The melanin present in their cell walls enhances resistance to host oxidative mechanisms and antifungal agents, thereby contributing to pathogenicity [3]. Clinically, the presence of brown or pigmented stromal infiltrates is considered suggestive of dematiaceous fungal infection, although this sign may be subtle and requires careful slit-lamp evaluation [4].

Topical corticosteroid use prior to microbiological confirmation has been associated with poorer outcomes in fungal keratitis [5]. Steroids suppress local immune responses, promote fungal proliferation, and may mask the severity of infection, thereby delaying appropriate treatment.

Before presenting at our center, the patient had been evaluated elsewhere and treated with topical corticosteroids for presumed pterygium-associated inflammation. Although the presence of a bifid pterygium and adjacent inflammation may have contributed to the initial clinical impression, corticosteroid use before microbiological confirmation can worsen fungal keratitis by suppressing local immune responses and promoting fungal proliferation. However, in the absence of meticulous corneal staining and careful slit-lamp evaluation to assess for subtle stromal infiltrates, an underlying infectious pathology may be overlooked [6]. The differential diagnosis of a localized corneal infiltrate adjacent to a pterygium includes bacterial keratitis, sterile inflammatory keratitis, marginal keratitis, and other filamentous fungal infections such as *Fusarium* and *Aspergillus *keratitis. Clinical distinction may be challenging, particularly when characteristic pigmentation is subtle or absent. Previous studies have shown that visible pigmentation is present in only a minority of cases, and its absence should not exclude the possibility of dematiaceous keratitis. Furthermore, it has been reported that significant stromal inflammation can mask or obscure brown pigmentation, making early diagnosis challenging [7]. Careful slit-lamp examination, early microbiological evaluation, and prompt initiation of topical natamycin in this case resulted in favorable clinical and visual outcomes.

## Conclusions

This case illustrates a rare and potentially misleading presentation of microbiologically confirmed *Curvularia* keratitis occurring adjacent to a bifid pterygium in the setting of prior topical corticosteroid use. The report underscores the importance of maintaining a high index of suspicion in cases of presumed pterygial inflammation, particularly following ocular trauma. Careful slit-lamp biomicroscopy, routine corneal staining, and timely microbiological evaluation are essential before initiating corticosteroid therapy. Furthermore, this case highlights that inadvertent steroid use may mask clinical signs and facilitate fungal proliferation. Early recognition and appropriate antifungal management can lead to favorable anatomical and visual outcomes, even in steroid-modified presentations.
